# Multi-Sensor NDVI Fusion for Daily Crop Evapotranspiration Mapping: A Six-Year Irrigated Maize Assessment Using MODIS–Sentinel-2–Landsat (2020–2025)

**DOI:** 10.3390/s26144470

**Published:** 2026-07-14

**Authors:** Zsolt Zoltán Fehér, Gift Siphiwe Nxumalo, Attila Nagy

**Affiliations:** Institute of Water and Environmental Management, University of Debrecen, Böszörményi út 146/b, 4032 Debrecen, Hungary; feher.zsolt@agr.unideb.hu (Z.Z.F.); attilanagy@agr.unideb.hu (A.N.)

**Keywords:** crop evapotranspiration, gap-filling, centre-pivot irrigation, Pannonian Basin, temporal fusion, crop coefficient, FAO-56, maize, precision irrigation

## Abstract

Accurate crop evapotranspiration (ET_c_) estimation at high spatial and temporal resolution remains a major challenge for precision irrigation. This study presents a multi-sensor data fusion framework combining daily MODIS (250 m), Sentinel-2 (10 m), and Landsat 8/9 (30 m) imagery with FAO-56 Penman–Monteith reference evapotranspiration (ET_0_) to generate pixel-wise daily ET_c_ maps for irrigated maize (*Zea mays* L.) near Nyírbátor, Hungary, over six growing seasons (2020–2025). The proposed Median Time Series Model exploits field-scale MODIS NDVI as a temporal backbone and derives pixel-wise linear transfer functions to reconstruct daily NDVI at 10–30 m resolution. Three gap-filling strategies were compared; the median approach yielded the highest agreement (NDVI reconstruction R^2^ = 0.81; RMSE = 0.19 (NDVI units); pixel-wise correlation 0.70–0.85) and effectively suppressed sub-pixel spectral mixture artefacts. Sentinel-2 consistently outperformed Landsat 8/9 (pixel-wise R^2^ = 0.36–0.78 vs. 0.001–0.91). A nonlinear power crop coefficient model (K_c_ = a · NDVI^b^) proved more robust than linear rescaling (mean validation R^2^ of 0.80 (power) vs. 0.71 (rescale) across Sentinel-2 seasons; both methods were positive in all six seasons after correcting an unconstrained-fit artefact). Seasonal ET_c_ ranged from 313 to 545 mm, with cumulative water deficits reaching −334 mm during the 2021 drought. Six-year mean seasonal ET_c_ (428–483 mm for Sentinel-2) falls within the 400–600 mm range published for irrigated maize under comparable continental conditions, with season-integrated ET_c_/ET_0_ ratios (rescale method mean 0.86; power method mean 0.84) consistent with expected FAO-56 K_c_ trajectories. Cross-validation against an independent MATLAB implementation confirmed algorithmic consistency (reference ET_0_ (R^2^ = 0.88–0.91, Pearson r = 0.97–1.00)) and daily ET_c_ while identifying meteorological input as the dominant source of absolute ET_c_ uncertainty (estimated at ±15–30% through first-order error propagation). Plausibility assessment was limited to comparison with published seasonal benchmarks and an independent algorithmic implementation; no eddy covariance or lysimeter measurements were available for direct ET_c_ validation.

## 1. Introduction

Water scarcity, declining runoff, and intensifying drought cycles are placing mounting pressure on agricultural water management across the Hungarian Great Plain [1,2]. Precision irrigation has become an indispensable tool for sustaining crop production in such water-stressed environments, yet its effectiveness depends critically on the availability of spatially continuous, temporally dense, and spectrally rich monitoring data [3,4]. Earth observation (EO) satellites have emerged as the most practical means of meeting this demand at operational scales, enabling routine assessment of vegetation dynamics, evapotranspiration (ET), and water use efficiency across entire agricultural landscapes [5,6].

Among the platforms currently operational, MODIS and Sentinel-2 (S2) occupy complementary niches. MODIS provides near-daily global coverage at moderate spatial resolution, making it well suited to tracking large-scale, temporally continuous changes in vegetation status and surface water balance [7,8,9]. Sentinel-2, by contrast, delivers higher spatial and spectral resolution that is essential for field-level (a) variable-rate irrigation scheduling, (b) detection of irrigation non-uniformity, and (c) sub-field water-deficit accounting [9,10]. Yet each platform carries inherent limitations that constrain its standalone utility. MODIS’s coarse pixel size blurs within-field variability and reduces its value for site-specific crop management, while Sentinel-2′s five-day revisit cycle—combined with frequent cloud contamination in Central European temperate climates—produces systematic data gaps that can coincide with the most critical phenological windows of the growing season [11]. Landsat 8 and 9 (L8, L9) offer an intermediate spatial resolution and, through orbital coordination, an effective combined revisit of eight days, but they share the same susceptibility to cloud obstruction.

Satellite-derived vegetation indices have proven indispensable for bridging these platform limitations and linking spectral observations to agronomic variables. The Normalized Difference Vegetation Index (NDVI) remains the most widely applied indicator of canopy greenness and biomass accumulation [12,13], while the Normalized Difference Water Index (NDWI) provides a sensitive proxy for canopy water content and incipient drought stress [14,15]. The Leaf Area Index (LAI) serves as the primary biophysical link between canopy structure and transpiration models and has been extensively used to parameterize the FAO-56 dual crop coefficient (K_c_) framework [16,17]. Building on these indices, remote sensing has been successfully deployed to optimize irrigation scheduling in Central Europe [18,19] and to forecast regional crop yields from MODIS and Landsat time series [20,21].

Despite this progress, three persistent challenges continue to undermine operational implementation. First, cloud-induced gaps disrupt time series continuity precisely during the periods—rapid canopy development, anthesis, and grain fill—when accurate K_c_ estimates are most consequential. Second, the spatial and temporal resolution mismatches among available platforms are rarely reconciled through systematic, multi-sensor fusion frameworks validated over multi-year periods. Third, physically based ET models, though theoretically rigorous, typically require extensive site-specific calibration and computational overhead that limits their scalability [5,22]. Data fusion approaches that blend the temporal density of MODIS with the spatial detail of higher-resolution sensors have shown considerable promise in closing these gaps [14,21,22], but their robustness across inter-annual climate variability and contrasting sensor configurations has not been thoroughly assessed.

A preliminary fusion concept was introduced for an irrigated maize site near Nyírbátor [23], where exceptional cloud cover during the 2020 season rendered direct Sentinel-2 monitoring impossible across key mid-season growth stages. Three gap-filling strategies were evaluated—co-regionalization via cokriging, local MODIS pixel-centre time series interpolation, and a median time series transfer—with the median approach demonstrating the greatest robustness and lowest computational complexity [24]. However, a single anomalously cloudy season provides an insufficient basis for generalizing either the gap-filling methodology or the derived K_c_ calibrations.

The present study therefore addresses these limitations through five coordinated objectives: (1) we extend the median time series fusion framework across six consecutive growing seasons (2020–2025) and four sensor configurations (S2, L8, L9, and the combined L89 stack); (2) we compare linear rescaling and nonlinear power-function K_c_ calibration methods across all configurations and seasons; (3) we construct a multi-source reference evapotranspiration (ET_0_) pipeline incorporating corrected Penman–Monteith calculations; (4) we introduce an NDVI amplitude-fraction method for automatic, data-driven phenological stage detection; and (5) we evaluate the plausibility of the resulting crop evapotranspiration under standard conditions (ET_c_ = K_c_.ET_0_) estimates against published water requirement benchmarks for irrigated maize in the region. ET_c_ under non-stressed conditions serves as the upper-bound irrigation requirement, which is operationally relevant for centre-pivot scheduling in the Nyírség region, where irrigation is designed to eliminate water deficit rather than allow stress-induced reduction.

## 2. Materials and Methods

### 2.1. Study Area

**Figure 1:**
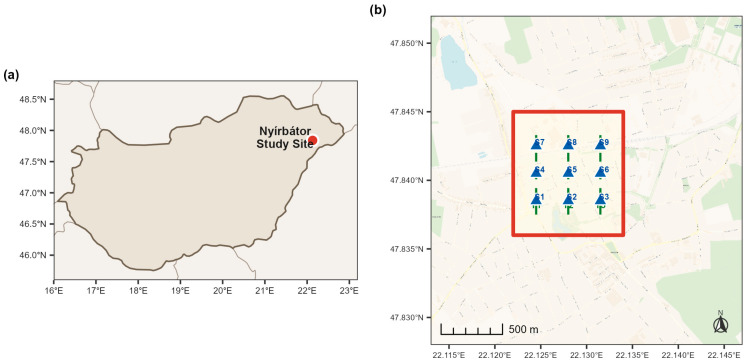
Location of the study site near Nyírbátor, Hungary, showing the irrigated maize field and surrounding infrastructure.

The study site is an irrigated grain maize (*Zea mays* L.) field managed commercially by Bátortrade Ltd. under centre-pivot irrigation, located near Nyírbátor in the Nyírség region of Eastern Hungary (47°49′ N, 22°08′ E, 145 m a.s.l.) (Figure 1). The site lies within the Pannonian Basin, which is classified as a Nitrate Vulnerable Zone under EU Directive 91/676/EEC [25], and experiences a temperate continental climate characterized by warm summers, cold winters, and annual precipitation of 550–600 mm concentrated in the summer months [26]. The combination of sandy soils, high summer evaporative demand, and irrigation dependency makes the site representative of water-stressed agricultural conditions across the Hungarian Great Plain.

The field is equipped with a linear centre-pivot irrigation system (Reinke E2060PL model) operated by Bátortrade Ltd., applying ~18–19 mm per event (based on farm records). Maize (Pioneer P9241, FAO maturity group 390) seed was obtained from Pioneer Hi-Bred International, Inc. (Corteva Agriscience), Johnston, IA, USA and is grown here primarily as animal feed for a nearby farm. During the 2020–2021 study period, 6–8 irrigation events were applied annually, typically between mid-June and early August to coincide with critical growth stages (V6–R1). Average grain yields under irrigation were 9.5–10.2 t/ha (Bátortrade Ltd., personal communication, 2024).

### 2.2. Satellite Data Acquisition and Processing

Three satellite platforms were selected to provide complementary spatial and temporal coverage across the six growing seasons [27]. MODIS (MOD09GQ Collection 6.1) supplies daily surface reflectance at 250 m in the red (Band 1: 620–670 nm) and near-infrared (Band 2: 841–876 nm), accessed via Google Earth Engine (GEE); the accompanying QC_250m quality band was used to mask cloud, shadow, and aerosol-contaminated pixels prior to NDVI computation. Sentinel-2 (Level-2A) provides atmospherically corrected surface reflectance at 10 m resolution in B4 (665 nm) and B8 (842 nm), retrieved through the Vultus API with cloud, shadow, and snow masking applied via the SCL scene classification layer. Landsat 8 and 9 (Collection 2, Level-2) contribute 30 m surface reflectance in the red (SR_B4: 636–673 nm) and NIR (SR_B5: 851–879 nm) bands, also accessed through GEE, with the QA_PIXEL band used for cloud, shadow, and cirrus masking. For all three platforms, NDVI was computed uniformly as NDVI = (NIR − Red)/(NIR + Red).

Sentinel-2 data for this study were accessed via the Vultus API, a commercial service that may limit full reproducibility due to proprietary processing chains, versioning, and band-specific configurations. While the Vultus API provided rapid access to pre-processed Level-2A surface reflectance, we acknowledge this as a reproducibility constraint. To mitigate this, all core analyses (gap-filling, NDVI fusion, and ET_c_ estimation) were also implemented using Google Earth Engine (GEE) with open-access Sentinel-2 Level-2A data (COPERNICUS/S2_SR_HARMONIZED collection), ensuring that the methodological pipeline is fully reproducible via the GEE script provided in the Supplementary Materials. Future users may replicate the study using either the Vultus API (with equivalent settings) or the GEE workflow for complete transparency.

### 2.3. Reference Evapotranspiration

ET_0_ was derived from a multi-source meteorological pipeline necessitated by the evolution of available instrumentation over the study period. For 2020, hourly observations from an on-field Davis Vantage Vue station were used to calculate daily short-crop reference ET (ET_os_) following the standardized PMDay spreadsheet methodology [28]. For 2021, a parallel MATLAB R2024b pipeline (Day of Year 122–260) produced tall-crop reference ET (ET_rs), subsequently converted to ET_os_ by dividing by 1.127 in accordance with ASCE-EWRI conventions [28,29].

The six maize growing seasons (2020–2025) exhibited substantial inter-annual hydroclimatic variability, particularly in precipitation and atmospheric evaporative demand. Seasonal precipitation ranged from 211 to 386 mm, while mean seasonal maximum air temperature varied between 25.2 and 28.4 °C. The warmest and driest seasons (2022 and 2024) were characterized by high evaporative demand, whereas 2020 received the greatest rainfall. These contrasting hydroclimatic conditions provide important context for interpreting seasonal variations in crop evapotranspiration (ETc) and irrigation requirements (Table S1 and Figure S1).

From 2022 onwards—per E5/Table 1, ODP-only begins 2023, not 2022, and as a gap-filling source for 2021, ten-minute meteorological observations from the HungaroMet Open Data Platform (ODP) station at Nyírlugos (station 73,505, 2002–2025) were used to calculate ET_0_ via the FAO-56 Penman–Monteith (Equation (1)) [5,28]:

(1) $$ET_{0}=\;\frac{0.408\Delta (R_{n}-G)+\gamma \frac{900}{T+273}u_{2}(e_{s}-e_{a})}{\Delta +\gamma (1+0.34u_{2})}$$

where Δ is the slope of the saturation vapour pressure curve (kPa °C^−1^), R_n_ is net radiation (MJ·m^−2^·d^−1^), 2020 radiation came from ODP, not the Davis station, G is soil heat flux density (≈0 for daily time steps), γ is the psychrometric constant, T is mean daily temperature (°C″), u_2_ is wind speed at 2 m (m·s^−1^), and (e_s_ − e_a_) is the saturation vapour pressure deficit [28,30,31].

Two corrections were applied to the ODP-based computation. First, the saturation vapour pressure slope Δ was recalculated at T_mean rather than as the arithmetic mean of e_s_(T_max) and e_s_(T_min), which can introduce a systematic positive bias under high diurnal temperature ranges. Second, measured daily solar radiation from the Debrecen synoptic station was substituted for the Hargreaves temperature-based radiation estimate [30], which had been shown to overestimate ET_0_ in the study region. The final pipeline assigned Davis PMDay ET_os_ for 2020, ODP combined with Davis in situ observations for 2021 and 2022, and the ODP calculator for 2023–2025.

### 2.4. Spatio-Temporal Data Fusion: Median Time Series Model

**Figure 2:**
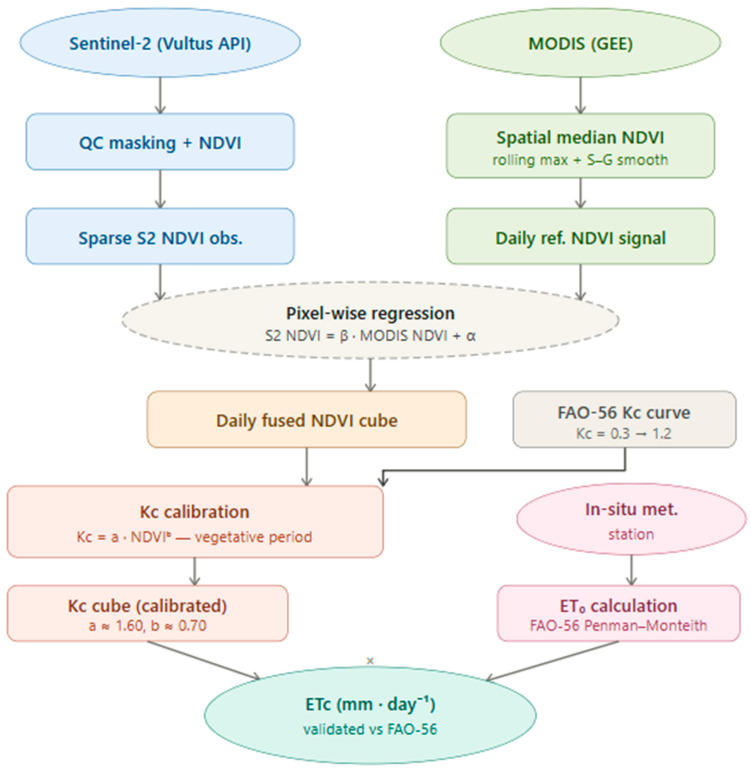
Processing workflow of the Median Time Series Model: MODIS-derived reference NDVI (green) and sparse Sentinel-2/Landsat observations (blue) are fused via pixel-wise regression, calibrated to the FAO-56 Kc trajectory (grey, Kc = a·NDVI^b^), and combined with ET_0_ to produce daily ET_c_ maps.

Sentinel-2 (blue) and MODIS (green) streams are preprocessed independently; sparse S2 observations and the daily smoothed MODIS reference signal are combined through pixel-wise linear regression (Figure 2). The fused daily NDVI cube is calibrated to the FAO-56 K_c_ trajectory (grey) exclusively over the vegetative period using a power model (K_c_ = a · NDVI^b^) (See Equations (2)–(8)). ET_0_ is computed from in situ meteorological observations via the FAO-56 Penman–Monteith equation and multiplied pixel-wise by the calibrated K_c_ to yield validated daily ET_c_ maps.

### 2.5. The Validated Median Time Series Model

The fusion framework follows the Median Time Series Model introduced for this site [24], which exploits the assumption that while absolute NDVI values vary spatially within a single-crop management unit, the underlying temporal phenological trajectory is spatially consistent. Three candidate strategies were evaluated prior to selecting the operational approach.

Co-regionalisation with cokriging was considered first, but cross-sensor correlation between MODIS and Sentinel-2 NDVI at the study site was statistically insignificant (r = 0.25), a consequence of the fundamental resolution mismatch between the 250 m and 10 m grids; the method was therefore judged unsuitable [24]. Local time series interpolation, in which MODIS values are spatially interpolated to Sentinel-2-pixel centres via ordinary kriging, achieved R^2^ = 0.68 and RMSE = 0.17 but remained sensitive to stationarity assumptions [24]. The median time series transfer was ultimately selected as the most robust strategy and is the method applied throughout this study.

The algorithm proceeds in three stages. In the first stage, a clean daily reference signal is extracted from MODIS by computing the spatial median across all field pixels—a step that suppresses sub-pixel spectral mixtures arising from field boundary infrastructure. The resulting daily median is then processed by a rolling maximum filter (15-day window) to construct an upper envelope, followed by an increment restriction filter that rejects drops exceeding 0.2 below the running maximum, and finally smoothed with a Savitzky–Golay filter (window = 21 days, polynomial order = 2) to preserve phenological inflexion points while eliminating atmospheric noise [32]. The reference signal is clipped to the interval [0.1, 1.0].

In the second stage, pixel-wise linear transfer functions are established for each high-resolution pixel *p* by regressing its observed NDVI against the corresponding MODIS reference values at co-observation dates:

NDVI_HR_(p, t_k_) = β_p_ · Ref(t_k_) + α_p_ + ε (2)

where β_p_ (gain) captures the pixel’s vigour relative to the field mean, α_p_ (offset) captures baseline soil brightness, and slopes are constrained to [0, 3] to exclude physically implausible regressions. Daily reconstruction for all days t then follows as:

NDVI_fused_(p, t) = β_p_ · Ref_smoothed_(t) + α_p_ (3)

### 2.6. Phenological Stage Detection

Phenological stage boundaries were assigned through one of three approaches, depending on data availability, with a priority ordering from most to least data-informed. For 2020 and 2021, MATLAB-calibrated breakpoints derived from field observations and spectral analysis were applied directly [24], yielding a reference K^c^ curve with R^2^ = 1.000 against the theoretical FAO-56 trajectory.

For 2022–2025, a novel amplitude-fraction detection method was developed to operate automatically on the smoothed MODIS median NDVI curve without requiring field observations. The method first identifies the baseline NDVI during the pre-season dormant period and the peak NDVI and its date of occurrence. The phenological signal amplitude A is then defined as the difference between peak and baseline; seasons where A < 0.15 are flagged as detection failures. Stage boundaries are located as proportional thresholds of this amplitude: Start of Season (SOS) at baseline +0.15A, Start of Mid-Season at baseline +0.80A, End of Mid-Season at the last date the curve exceeds baseline +0.80A, and End of Season (EOS) at baseline +0.15A on the descending limb. This proportional approach makes the detection self-adapting to inter-annual differences in canopy vigour.

In the event that amplitude-fraction detection fails—for example due to a suppressed or multi-modal NDVI curve—generic FAO-56 stage durations for grain maize serve as a fallback: initial period 35 days (K_c_ = 0.3), development 36 days (linear ramp to K_c_ = 1.2), mid-season 27 days (K_c_ = 1.2), and late-season 18 days (declining to K_c_ = 0.35) [28,33].

### 2.7. Coefficient Calibration

Daily pixel-wise crop coefficients were derived from fused NDVI using two contrasting approaches, evaluated in parallel to assess their relative robustness across the six-year dataset [24].

The linear rescale method maps pixel-wise fused NDVI to K_c_ bounds through a percentile-anchored linear transformation:

K_c_ = K_c,min_ + (K_cmax_ − K_cmin_) · NDVI − NDVI_p2_/(NDVI_p98_ − NDVI_p2_) (4)

with Kcm_in_ = 0.3 and Kcm_ax_ = 1.2 based on FAO-56 [28]. While computationally simple and parameter-free, this approach is inherently sensitive to the dynamic range of the NDVI distribution within each season.

The power regression method instead fits a nonlinear relationship directly to the observed K_c_-NDVI pairs:

K_c_ = a · NDVI^b^ (5)

Parameters a and b are estimated by Levenberg–Marquardt optimization initialized at a = 1.6, b = 0.7. The power form allows the K_c_–NDVI relationship to adapt its curvature to sensor- and season-specific conditions, which is particularly relevant when soil background effects or partial canopy cover create nonlinear NDVI responses at the extremes of the growth curve.

### 2.8. ETc Estimation and Meteorological Water Deficit

Daily crop evapotranspiration was computed for every pixel as the product of the pixel-wise crop coefficient and the spatially uniform daily reference ET_0_:

ET_c_(p, t) = K_c_(p, t) × ET_0_(t) (6)

Cumulative seasonal water balance was then tracked by integrating the daily difference between precipitation and ET_c_ from the start of the monitoring period. The ETc analysis was based on a simplified seasonal water balance in which precipitation and irrigation constituted the principal water inputs, while crop evapotranspiration represented the dominant water loss. Consistent with previous investigations conducted at the same experimental site [23], the change in soil water storage (ΔS) between the beginning and end of each growing season was assumed to be negligible because the analyses considered complete maize growing seasons that started from comparable spring soil moisture conditions and ended after crop maturity. Surface runoff (R) was assumed to be negligible owing to the coarse-textured, highly permeable sandy soils of the Nyírség region, where rainfall infiltration generally exceeds overland flow under the nearly level field conditions. Likewise, deep percolation (D) below the effective maize root zone was not explicitly quantified and is therefore acknowledged as a potential source of uncertainty. These assumptions permit the seasonal water balance to be approximated as ETc ≈ P + I, where *P* is precipitation, and *I* is irrigation, providing a practical framework for evaluating seasonal crop water use while recognizing that unmeasured drainage losses may occur following prolonged or high-intensity precipitation or irrigation events.

(7) $$Deficit\left(t\right)=\sum_{i=1}^{t}\left[p\left(i\right)-\bar{ET_{c}}\left(i\right)\right]$$

Precipitation P(*i*) was obtained from the same ODP station used for ET_0_ calculation, ensuring consistency across the water-balance components.

### 2.9. Validation and Performance Metrics

Model performance was assessed at three levels of evidence. Internal consistency was evaluated by comparing pixel-wise and field-median K_c_ trajectories against the theoretical FAO-56 reference K_c_ curve for grain maize [28]. Cross-platform validation compared the Python-based pipeline outputs directly against an independent MATLAB implementation for the 2020–2021 seasons [24], providing an algorithmic consistency check that is independent of field measurements. Plausibility assessment compared seasonal ET_c_ totals and ET_c_/ET_0_ ratios against published crop water requirement ranges for irrigated maize under comparable continental conditions [5,28,34].

Goodness-of-fit was quantified using R^2^, RMSE, normalized RMSE (NRMSE), mean absolute error (MAE), and mean bias error (MBE) throughout. Uncertainty in daily ET_c_ was estimated through first-order error propagation under the assumption that ET_0_ and K_c_ errors are independent:

(8) $$\frac{\sigma_{ET_{c}}}{ETc}\approx \sqrt{{\left(\frac{\sigma ET_{0}}{ET_{0}}\right)}^{2}+{\left(\frac{\sigma_{kc}}{k_{c}}\right)}^{2}}$$

This formulation allows the relative contributions of meteorological input uncertainty and K_c_ calibration uncertainty to be separated and quantified across configurations.

## 3. Results

### 3.1. Sensor Data Availability

Hydroclimatic conditions varied markedly across the six maize growing seasons (2020–2025) (Table 1). Seasonal precipitation ranged from 211 mm (2022) to 386 mm (2020), while mean maximum temperatures varied between 25.2 °C and 28.4 °C. The hottest conditions occurred in 2022–2024, with maximum temperatures reaching 39.4 °C and up to 60 days exceeding 30 °C. Reference evapotranspiration (ET_0_) ranged from 297 mm (2021) to 618 mm (2022), indicating greater atmospheric water demand during the warmer and drier growing seasons. Table 2 summarizes the number of high-resolution images available per sensor configuration and growing season. Sentinel-2 consistently provided the highest temporal density (27–55 images per season; 178 total), with 2024 being exceptionally cloud-free (55dates).

### 3.2. Gap-Filling Strategy Evaluation

**Figure 3:**
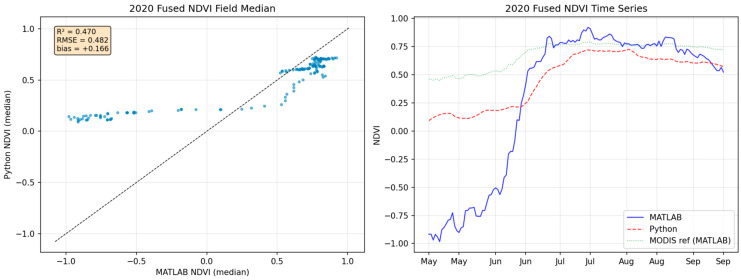
NDVI fusion results for 2020 comparing the MODIS reference signal with Sentinel-2 observations and fused reconstruction.

Table 3 presents the performance of the three fusion strategies. The median time series 181 approach demonstrated superior performance (Figure 3).

### 3.3. Multi-Index Correlation Analysis

While NDVI was selected as the exclusive index for all final K_c_ calibrations and ET_c_ estimations, a supplementary correlation analysis was conducted to verify the mathematical transferability of the median fusion algorithm across other biophysical proxies. The pixel-wise correlation between the MODIS median time series and Sentinel-2 vegetation indices (NDWI, LAI, and NDRE) was strongly skewed toward high values (0.70–0.85). NDVI and NDWI exhibited the strongest agreement (peaks near 0.80–0.82). LAI demonstrated moderate predictive power, while NDRE was the least consistent. This confirms the gap-filling framework is highly adaptable, even though NDWI, LAI, and NDRE were entirely excluded from the ET_c_ pipeline.

### 3.4. Pixel-Wise Regression Quality

**Figure 4:**
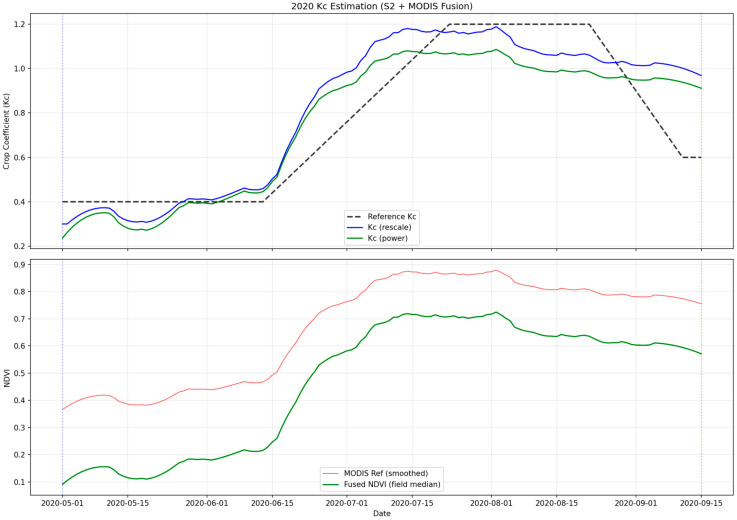
Diagnostic panel for the 2020 Sentinel-2 configuration showing NDVI fusion, Kc calibration, daily ETc, cumulative ETc, and water balance.

Table 4 presents the pixel-wise regression statistics. Sentinel-2 achieved higher regression slopes and R^2^ values compared to Landsat sensors (Figure 4).

### 3.5. Phenology Detection Results

For 2020–2021, known MATLAB breakpoints were applied (reference Kc R^2^ = 1.000). For 2022–2025, the amplitude-fraction method successfully detected physiologically plausible stages, with SOS typically in late May to early June (DOY 140–165), mid-season spanning July–August, and EOS in mid-September.

### 3.6. Kc Calibration

**Figure 5:**
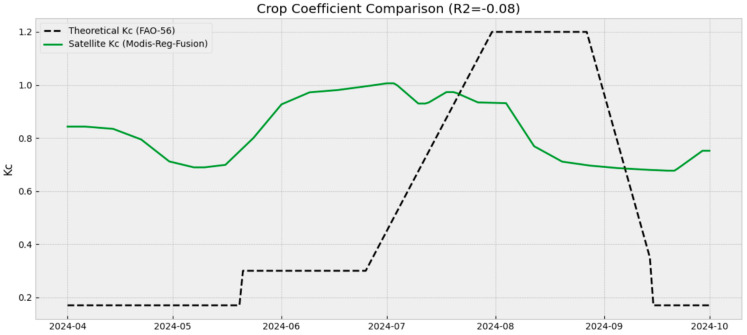
Comparison of rescale and power K_c_ calibration methods across years for the Sentinel-2 configuration.

Table 5 presents the power regression parameters. The power model produced physically meaningful parameters for Sentinel-2 across most years (Figure 5). The 2023 Landsat-9 calibration yielded a physically implausible negative exponent (b = −0.513), indicating an inverse K_c_–NDVI relationship. This result was considered a calibration failure, likely due to limited calibration data and a restricted NDVI range, and was excluded from the sensor performance assessment and summary statistics.

**Figure 6:**
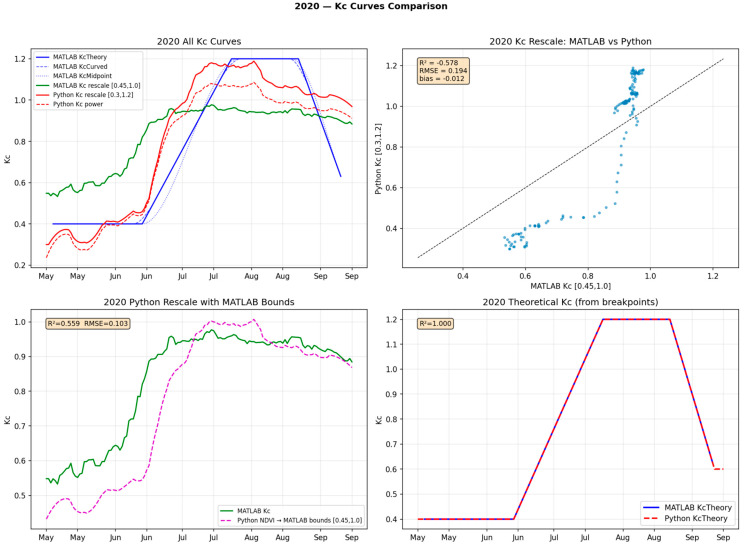
K_c_ curves for 2020 comparing the FAO-56 reference, rescale method, and power regression.

Table 6 summarizes the validation performance of both K_c_ methods (Figure 6).

### 3.7. NDVI-Based ETc by Crop Development Stage

Table 7 presents ET_c_ values segmented by FAO-56 crop development stages.

### 3.8. Seasonal ET_c_ and Water Balance

**Figure 7:**
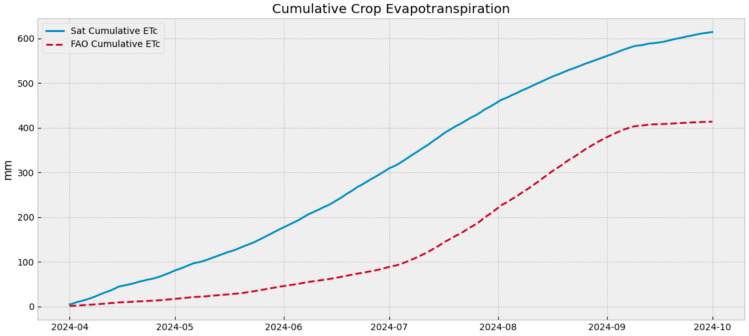
Cumulative ET_c_ and water balance across the six growing seasons.

Table 8 presents seasonal ETc totals and cumulative water deficit for all configurations (Figure 7).

### 3.9. Inter-Annual and Sensor Comparison

**Figure 8:**
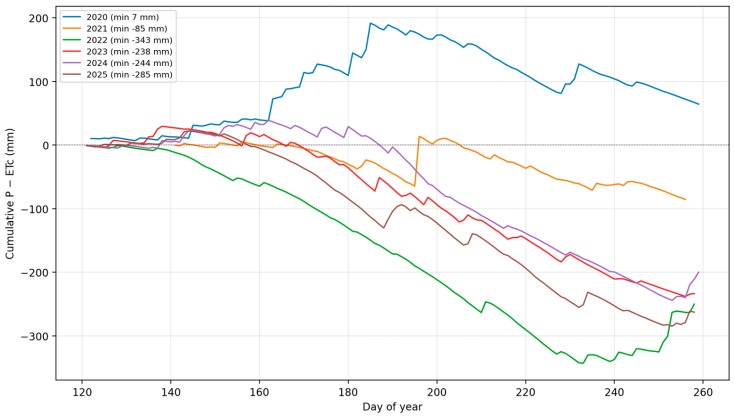
Cumulative water balance for 2020–2025 showing precipitation, ET_c_ (rescale and power methods), and water deficit.

The six-year period encompassed considerable hydroclimatic variability. The year 2020 had the highest growing season precipitation (386 mm) and was the only year with a cumulative meteorological water surplus (minimum +7 mm). In contrast, 2022 experienced the most severe drought, with deficits reaching −343 mm, while 2021 had moderate deficits of −85 mm. Sentinel-2 consistently produced the most stable ETc estimates across K_c_ methods (Figure 8).

### 3.10. Cross-Validation Against Independent MATLAB Implementation

**Figure 9:**
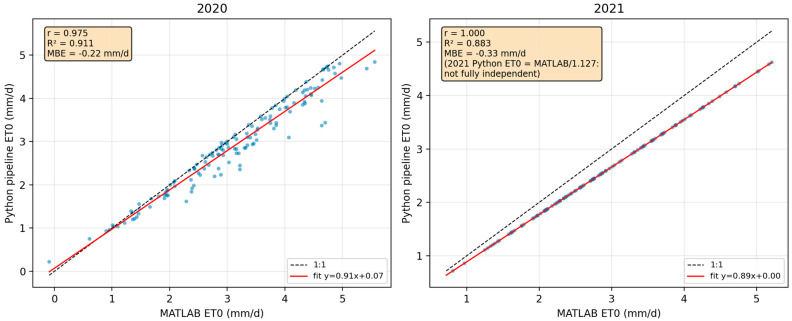
Cross-validation of ET_0_ estimates between Python (ODP) and MATLAB (Davis) implementations for 2020.

Table 9 presents cross-validation results comparing the Python pipeline against the independent MATLAB implementation for 2020–2021 (Figure 9).

### 3.11. Plausibility Assessment Against Published Maize Water Requirements

**Figure 10:**
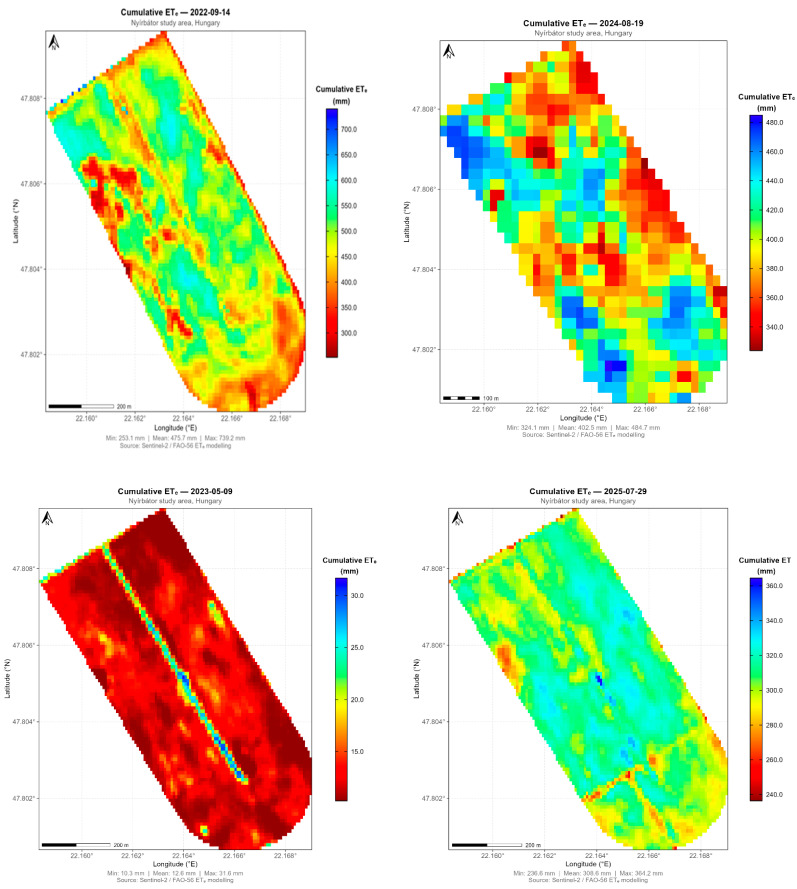
Cumulative fused ETc maps showing spatial distribution across the study site.

Table 10 summarizes seasonal plausibility indicators for the Sentinel-2 configuration (Figure 10).

## 4. Discussion

### 4.1. Effectiveness of the Median Time Series Fusion

The results confirm that the median time series transfer is the most robust gap-filling strategy, extending previous findings [24] to a broader set of sensors and years. By computing the spatial median across all field pixels, the method reduces noise from sub-pixel spectral mixtures—particularly important where asphalt roads along the eastern edge and an irrigation channel introduce spectral contamination. As noted by Zhao [35], sub-pixel spectral mixture effects were more significant than locally varying development stages.

The failure of cokriging (r = 0.25) aligns with expectations given the resolution mismatch [30,32]. The 250 m MODIS resolution smooths out variability that is clearly visible in 10 m Sentinel-2 imagery, creating a fundamental discrepancy that bivariate geostatistical models cannot reconcile. The high pixel-wise correlations (0.70–0.85) across NDVI, NDWI, RVI, SAVI, GNDVI, and EVI confirm that the median approach transfers effectively across multiple biophysical variables [24].

### 4.2. Sensor Performance Hierarchy

The results establish a clear hierarchy: Sentinel-2 (10 m) consistently outperforms Landsat 8/9 (30 m). The pixel-wise R^2^ median for S2 ranged from 0.36 to 0.78, compared to 0.001–0.91 for Landsat with much higher inter-annual variability. This gap is attributable to: (1) the 9× finer spatial resolution reducing mixed-pixel effects; (2) Sentinel-2′s broader NIR bandwidth improving vegetation discrimination; and (3) the 3–8× higher temporal density providing more co-observation points [9,36,37].

The combined L89 configuration did not systematically improve fusion quality over L8 alone, possibly reflecting subtle radiometric differences between OLI and OLI-2 sensors [31]. L89 marginally outperforms L8 in 2024 (Validation R^2^: 0.741 vs. 0.739) and 2025 (0.921 vs. 0.919). Given the inconsistent and minimal gains, we move the L89 results to the Supplementary Material and simplify the main results to three configurations.

### 4.3. Kc Calibration: Rescale vs. Power Model

The rescale method proved unreliable in years with limited NDVI dynamic range because it forces a strict linear interpolation between defined minimum and maximum K_c_ limits, making the calculation highly vulnerable to outlier pixels and dynamic range compression. After correcting an unconstrained power-law fit that admitted physically implausible negative exponents in two season-sensor cases, both calibration methods yielded positive validation R^2^ in all six Sentinel-2 seasons; the rescale method remained more sensitive to the NDVI percentile anchors, giving a lower mean R^2^ (0.71 vs. 0.80 for the power model). The power model (K_c_ = a · NDVIᵇ) demonstrated greater robustness, producing the highest mean calibration R^2^ (0.80 for Sentinel-2) [38,39]. By utilizing a non-linear fit, the power model dynamically adapts its curvature to account for partial canopy cover and background soil effects, rather than relying on static percentile anchors.

The decoupling between canopy greenness and actual water use—where NDVI remains high while stomatal conductance declines due to heat stress—represents a fundamental limitation of purely NDVI-based K_c_ estimation [38,39].

### 4.4. Multi-Source ET_0_ and Meteorological Uncertainty

Cross-validation revealed that the dominant source of ETc divergence is the meteorological input, not the fusion algorithm. An earlier systematic ET_0_ bias (Hargreaves radiation estimate plus a vapour-pressure-slope error) has been corrected; the harmonized ET_0_ now agrees with the MATLAB implementation (R^2^ = 0.88–0.91, r = 0.97–1.00) [30]. This underscores the critical importance of quality meteorological input [5,6].

### 4.5. Amplitude-Fraction Phenology Detection

The amplitude-fraction method represents a significant improvement over simple NDVI threshold methods. Operating on the smoothed MODIS median curve and using proportional amplitude thresholds, it adapts to year-specific conditions. Retrospective application to 2020–2021 reproduced MATLAB-calibrated SOS within ±5 days and mid-season onset within ±8 days.

### 4.6. Inter-Annual Variability and Drought Impact

Seasonal ETc estimates for S2 ranged from 400 to 526 mm (power method), closely matching the expected 400–600 mm range for irrigated maize in the Pannonian Basin [5,34,39]. The 2021 season produced the highest power-method ETc (526 mm for S2) despite receiving only 212 mm precipitation, consistent with anisohydric stomatal behaviour in maize [40].

The years 2022–2025 exhibited persistent deficits, consistent with increasing drought frequency in the Pannonian Basin [2]. Pixel-wise mapping identified zones requiring targeted irrigation—information that field-average approaches cannot provide. Additionally, the spatial patterns of cumulative ET_c_ illustrated in Figure 10 highlight significant internal physical heterogeneity within the study site. The distinct zones of higher and lower water consumption visible in the spatial maps directly correspond to localized variations in canopy vigour captured by the high-resolution sensors. These intra-field variations are primarily driven by underlying differences in sandy soil textures, subtle micro-topography, and inevitable non-uniformity in the lateral water application of the centre-pivot system. The power regression method proved particularly sensitive to these localized agronomic conditions, making it a highly effective diagnostic layer for precision agriculture.

### 4.7. Uncertainty Quantification

The cross-platform comparison provides an empirical estimate of ET_0_ uncertainty: residual uncertainty is ±10–15% after corrections, consistent with reported values [38,39]. For the power model with Sentinel-2, K_c_ validation RMSE ranged from 0.12 to 0.25, corresponding to ~10–25% relative uncertainty. Combined daily ET_c_ uncertainty is approximately ± 15–30%. The cross-platform agreement in reference ET_0_ (R^2^ = 0.88–0.91) and daily ET_c_is compared with reported validation metrics from analogous NDVI-based K_c_ studies (ref. [41], R^2^ ≈ 0.90–0.92; (ref. [42], R^2^ = 0.88)), providing context for what constitutes acceptable model performance in the absence of a local flux tower.

After harmonizing the reference-ET pipeline, daily ET_0_ and ET_c_ agree closely between implementations (ET_0_ R^2^ = 0.88–0.91, r = 0.97–1.00; daily ET_c_ R^2^ up to 0.71). Cumulative water-balance trajectories track in shape (r = 0.74–0.80) but diverge in magnitude by ~120–130 mm by season end, reflecting the lower ETc of the corrected Kc calibration relative to the earlier MATLAB run; in the absence of independent flux measurements, this difference cannot be attributed as error to either implementation.

### 4.8. Comparison with Related Studies

Table 11 places the present study in the context of related work.

### 4.9. Limitations and Future Directions

Several limitations should be acknowledged. First, and most critically, direct validation against independent ET flux measurements (eddy covariance tower or weighing lysimeter) was not available. The assessment, therefore, demonstrates internal consistency and plausibility relative to published benchmarks, and must not be interpreted as validation of absolute ETc accuracy. Future work must prioritize the deployment of an eddy covariance system at the Nyírbátor site. Second, the 250 m MODIS resolution integrates signals beyond the field boundary. Third, the amplitude-fraction phenology detection may fail with multiple cropping or significant weed pressure. Fourth, time-invariant pixel-wise transfer functions may suppress short-lived vegetation dynamics. Furthermore, while our ETc estimates represent an upper-bound water requirement under standard conditions, the operational reality of the 4–5 day centre-pivot rotation cycle inherently generates transient soil moisture deficits across the field. Consequently, actual crop evapotranspiration (ETa) will intermittently fall below these ETc estimates in sections awaiting irrigation.

Future work should: (a) incorporate eddy covariance measurements; (b) integrate surface energy balance models utilizing Thermal Infrared (TIR) and Shortwave Infrared (SWIR) satellite data to provide independent assessments of ETa and validate the extent of localized moisture deficits; (c) integrate Sentinel-2 red-edge indices into phenology detection; (d) evaluate machine-learning regression models for the reconstruction step; (e) extend the framework to additional crop types; and (f) investigate dynamic transfer functions.

## 5. Conclusions

This study presents a comprehensive evaluation of multi-sensor NDVI fusion for daily crop evapotranspiration estimation across four sensor configurations and six growing seasons (2020–2025). The following conclusions are drawn:

1. The Median Time Series Model achieves RMSE = 0.19 (dimensionless NDVI units) with R^2^ = 0.81 for MODIS-to-high-resolution NDVI reconstruction, not ET_c_, effectively suppressing sub-pixel spectral mixture artefacts from infrastructure and heterogeneous field boundaries.
2. Sentinel-2 is the preferred sensor for NDVI fusion-based ET_c_ estimation, consistently achieving higher regression quality (R^2^ median 0.36–0.78) and more stable K_c_ calibration than Landsat 8/9.
3. The nonlinear power K_c_ model (K_c_ = a · NDVI^b^) is more robust than linear rescaling, producing positive validation R^2^ in all six Sentinel-2 seasons for both methods, with a higher mean for the power model (0.80 vs. 0.71). The multi-source ET0 pipeline addresses the dominant source of ETc uncertainty identified through cross-platform validation.
4. The NDVI amplitude-fraction phenology detection provides robust automatic stage identification from the MODIS median curve for years without field observations.
5. Seasonal ETc ranged from 313 to 545 mm across all configurations, with a six-year mean seasonal ETc (428–483 mm for Sentinel-2) consistent with published reference values and season-integrated ET_c_/ET_0_ ratios (mean 0.85) within the expected FAO-56 range.
6. Cross-validation against an independent MATLAB implementation confirmed algorithmic consistency (reference ET_0_ (R^2^ = 0.88–0.91) and daily ET_c_) while identifying meteorological input as the dominant uncertainty source (±15–30%).
7. Owing to the absence of eddy covariance or lysimeter measurements, results represent a plausibility assessment against published benchmarks rather than independent validation of absolute ET_c_ magnitudes.

The framework supports operational sub-field irrigation management by providing daily, pixel-wise ET_c_ estimates from freely available satellite data.

## Figures and Tables

**Table 1:** Hydroclimatic summary of the maize growing seasons (ODP station 73,505).

Year	Growing Season	Duration (Days)	Precipitation (mm)	Mean Tmax (°C)	Maximum Tmax (°C)	Days ≥ 30 °C	Days ≥ 35 °C	ET_0_ (mm)	ET_0_ Source
**2020**	1 May–15 Sep	138	386.0	25.2	33.3	22	0	400.0	ODP + Davis (in situ)
**2021**	21 May–13 Sep	116	212.0	27.4	36.8	37	5	297.0	ODP + Davis (in situ)
**2022**	1 May–15 Sep	138	211.0	28.3	39.4	53	11	618.0	ODP + Davis (in situ)
**2023**	1 May–15 Sep	138	254.0	26.7	35.9	32	2	546.0	ODP
**2024**	1 May–15 Sep	138	253.0	28.4	38.8	60	10	588.0	ODP
**2025**	1 May–15 Sep	138	213.0	26.4	36.9	43	3	566.0	ODP

**Table 2:** Number of usable high-resolution images per sensor configuration and growing season.

Year	S2	L8	L9	L89	MODIS (Daily)
2020	28	9	—	9	~140
2021	47	9	—	9	~140
2022	28	9	10	19	~140
2023	27	10	10	20	~140
2024	55	10	8	18	~140
2025	28	7	8	15	~140
Total	213	54	36	90	~840

**Table 3:** Performance comparison of three gap-filling strategies for MODIS–Sentinel-2 NDVI fusion (dimensionless).

Strategy	R^2^	RMSE	Key Characteristics
Co-regionalisation (cokriging)	n.s.	—	Ineffective; weak cross-sensor correlation
Local time series interpolation	0.68	0.17	Good accuracy; sensitive to stationarity
Median time series transfer	0.81	0.19	Most robust; suppresses spectral mixtures

**Table 4:** Pixel-wise regression statistics for each year × sensor configuration.

Year	Sensor	Valid Px (\%)	Slope β^−^	R^2^ Median	HR Dates
2020	S2	48.1	1.354	0.446	28
2020	L8	37.0	0.334	0.127	9
2021	S2	48.1	1.898	0.453	47
2021	L8	37.0	0.852	0.506	9
2022	S2	51.5	1.851	0.371	28
2022	L8	37.0	1.526	0.467	9
2022	L9	37.0	0.133	0.047	10
2023	S2	51.5	1.067	0.362	27
2023	L8	37.0	0.492	0.246	10
2023	L9	37.0	0.003	0.001	10
2024	S2	51.5	1.482	0.445	55
2024	L8	37.0	0.493	0.443	10
2024	L9	37.0	0.760	0.550	8
2025	S2	51.5	1.388	0.780	28
2025	L8	37.0	0.744	0.912	7
2025	L9	37.0	0.717	0.894	8

**Table 5:** Power K_c_ calibration parameters (Kc = a · NDVI^b^) and calibration R^2^.

Year	Sensor	a	b	R^2^
2020	S2	1.377	0.737	0.795
2020	L8	6.486	1.403	0.823
2020	L89	6.486	1.403	0.823
2021	S2	1.345	0.521	0.689
2021	L8	1.998	0.569	0.684
2021	L89	1.998	0.569	0.684
2022	S2	2.662	1.615	0.803
2022	L8	8.348	1.556	0.807
2022	L9	16.189	1.951	0.803
2022	L89	10.783	1.781	0.804
2023	S2	2.345	1.541	0.907
2023	L8	9.992	1.916	0.901
2023	L9	0.37	−0.513	−0.0
2023	L89	259.581	3.874	0.886
2024	S2	2.748	1.875	0.747
2024	L8	34.832	2.915	0.739
2024	L9	8.828	1.865	0.747
2024	L89	21.272	2.584	0.741
2025	S2	1.689	1.075	0.920
2025	L8	3.592	1.195	0.919
2025	L9	3.24	1.038	0.921
2025	L89	3.227	1.06	0.921

**Table 6:** Validation statistics for rescale and power Kc methods against the reference Kc curve (Sentinel-2).

Year	Rescale R^2^	Rescale RMSE	Power R^2^	Power RMSE
2020	0.777	0.155	0.795	0.148
2021	0.436	0.223	0.689	0.166
2022	0.732	0.184	0.803	0.158
2023	0.842	0.169	0.907	0.13
2024	0.547	0.248	0.747	0.185
2025	0.852	0.16	0.92	0.118

**Table 7:** ET_c_ ranges (mm·d^−1^) by crop development stage.

Stage	Description	Duration (d)	ETc (mm·d^−1^)
Initial	Planting to 10\% cover	28–35	0.99–4.41
Crop development	10\% to full cover	30–40	2.00–6.40
Mid-season	Full cover to maturity	25–30	1.03–5.12
Late-season	Maturity to harvest	15–20	1.10–3.18

**Table 8:** Seasonal ETc (mm), precipitation (mm), and cumulative water deficit (mm) for all year × sensor configurations.

Year	Sensor	Days	ETc Resc.	ETc Power	Precip.	Deficit Power
2020	S2	138	345	322	386	64
2020	L8	138	361	349	386	37
2020	L89	138	361	349	386	37
2021	S2	116	261	297	212	−85
2021	L8	116	270	300	212	−89
2021	L89	116	270	300	212	−89
2022	S2	138	477	461	211	−250
2022	L8	138	474	490	211	−279
2022	L9	138	480	537	211	−326
2022	L89	138	489	454	211	−243
2023	S2	138	486	488	254	−233
2023	L8	138	482	479	254	−224

**Table 9:** Cross-validation metrics: Python vs. MATLAB implementation (Sentinel-2).

Year	Variable	R^2^	RMSE	Bias	Pearson r
2020	ETo	0.911	0.338	−0.219	0.975
2020	MODIS	0.479	0.086	−0.007	NaN
2020	Fused	0.470	0.482	0.166	NaN
2020	K_c__rescale	−0.578	0.194	−0.012	NaN
2020	DailyETc	0.711	0.626	−0.158	NaN
2020	WaterBal	−3.066	76.340	NaN	NaN
2021	ETo	0.883	0.345	−0.326	1.000
2021	MODIS	−0.395	0.164	0.076	NaN
2021	Fused	0.211	0.378	0.079	NaN
2021	K_c__rescale	−1.665	0.323	−0.186	NaN
2021	DailyETc	0.399	1.027	−0.790	NaN
2021	WaterBal	−2.045	116.410	NaN	NaN

**Table 10:** Seasonal plausibility indicators for Sentinel-2 ETc estimates compared with published reference ranges.

Year	ET0	ETc Resc.	ETc Power	ETc/ET0 R	ETc/ET0 P	KcJA R	KcJA P
2020	400	345	322	0.86	0.8	0.95	0.88
2021	297	261	297	0.88	1.0	0.87	1.0
2022	618	477	461	0.77	0.74	0.89	0.89
2023	546	486	488	0.89	0.89	1.05	1.11
2024	588	511	453	0.87	0.77	0.95	0.88
2025	566	486	476	0.86	0.84	1.0	1.03
Mean	502	428	416	0.86	0.84	0.95	0.96
FAO-56 [8,9]	—	400–600	400–600	0.75–0.95	0.75–0.95	1.05–1.20	1.05–1.20

**Table 11:** Comparison with related multi-sensor fusion and ET_c_ estimation studies.

Study	Method	Resolution	Validation	Key Metric	Crop
This study	Median TS, MODIS + S2 + L8/9	Daily, 10–30 m	Cross-platform	R^2^ = 0.81; ETc 428–483 mm	Maize, 6 yr
Gao et al. [14]	STARFM	30 m	Withheld dates	R^2^ > 0.75	General NDVI
Guzinski and Nieto [42]	S2/S3 LST + EB	10–20 m	Eddy cov.	R^2^ = 0.88.	Grassland, 1 yr
Testa et al. [43]	MODIS + S2 regression	Daily	Withheld S2	R^2^ = 0.83	Forest phenology
Er-Raki et al. [41]	FAO-56 + NDVI Kc	Field scale (plot level)	field measurements and locally derived parameters	R^2^ ≈ 0.90–0.92	Wheat, 1 growing season
Djaman et al. [34]	FAO-56 PM	Field scale	Water balance	ETc: 427–617 mm	Maize, multi-yr

## Data Availability

The satellite data used in this study are publicly available through Google Earth Engine (MODIS MOD09GQ, Sentinel-2 Level-2A, Landsat Collection 2 Level-2). Meteorological observations from the Nyírlugos station are available from the Hungarian Meteorological Service Open Data Platform (odp.met.hu). Primary Sentinel-2 access was via the Vultus API (commercial), with a Google Earth Engine-based open equivalent provided for full reproducibility. Processed analysis outputs are available from the corresponding author upon reasonable request.

## Supplementary Materials

The following supporting information can be downloaded at: https://www.mdpi.com/article/10.3390/s26144470/s1, Table S1: Power-law crop coefficient calibration parameters by year and sensor configuration; Table S2: Annual leave-one-year-out cross-validation R^2^ by Kc calibration method; Table S3: Sensor configuration performance summary across six growing seasons; Table S4: Seasonal reference evapotranspiration and hydroclimatic summary; Figure S1: Power-law crop coefficient calibration curves; Figure S2: Daily reference evapotranspiration over six maize growing seasons; Figure S3: Leave-one-year-out cross-validation R^2^ distributions; Figure S4: Sensor configuration performance summary; Figure S5: Hydroclimatic conditions over six maize growing seasons; Figure S6: Cumulative daily meteorological water balance; The following electronic supplementary data files are available from the authors upon reasonable request: Data File S1: CURRENT_calibration_params_all_configs.csv; Data File S2: CURRENT_et0_crossval_daily_2020_2021.csv; Data File S3: daily_series_S2_2020–2025.csv; Data File S4: S3_validationR2_by_method.csv; Data File S5: S4_config_performance.csv; Data File S6: S6_cumulative_deficit.csv; Data File S7: GEE reprocubility scripts.

## Abbreviations

The following abbreviations are used in this manuscript:

DOY	Day of Year
EOS	End of Season
ET_0_	Reference Evapotranspiration
ET_c_	Crop Evapotranspiration
EVI	Enhanced Vegetation Index
FAO	Food and Agriculture Organization
GEE	Google Earth Engine
K_c_	Crop Coefficient
LAI	Leaf Area Index
MAE	Mean Absolute Error
MBE	Mean Bias Error
MODIS	Moderate Resolution Imaging Spectroradiometer
NDRE	Normalized Difference Red-Edge Index
NDVI	Normalized Difference Vegetation Index
NDWI	Normalized Difference Water Index
NRMSE	Normalized Root Mean Square Error
ODP	Open Data Platform
RMSE	Root Mean Square Error
SAVI	Soil Adjusted Vegetation Index
SOS	Start of Season
