# Supplementary material for: Multi-Sensor NDVI Fusion for Daily Crop Evapotranspiration Mapping: A Six-Year Irrigated Maize Assessment Using MODIS–Sentinel-2–Landsat (2020–2025)
Source: Sensors (Basel). 2026 Jul 14;26(14):4470. doi: 10.3390/s26144470 (PMC13416505; doi:10.3390/s26144470)
Supplement: Supplementary file 1 [file sensors-26-04470-s001.zip › sensors-4362312-supplementary.pdf]

Supplementary Materials

Journal: *Sensors* (MDPI) — Special Issue: Remote Sensing in Precision Agriculture

Title: Multi-Sensor NDVI Fusion for Daily Crop Evapotranspiration Mapping: A Six-Year Irrigated Maize Assessment Using MODIS–Sentinel-2–Landsat (2020–2025)

Authors: Zsolt Zoltán Fehér , Nxumalo Gift Siphiwe and Attila Nagy

Affiliation: Doctoral School of Food Sciences, University of Debrecen, Debrecen, Hungary

SUPPLEMENTARY TABLES

Table S1. Power-law crop coefficient calibration parameters ($Kc = a \cdot NDVI^b$) by year and sensor configuration for the corrected pipeline (positive-b constraint enforced for Sentinel-2 across all six growing seasons). Cross-validation R^2 and RMSE are derived from leave-one-year-out validation against in situ ETc observations. n = number of cloud-free fused-NDVI dates contributing to calibration per season.

Year	Sensor	a	b	Validation R^2	RMSE	n
2020	S2	1.377	0.737	0.795	0.148	28
2020	L8	6.486	1.403	0.823	0.137	9
2020	L89 †	6.486	1.403	0.823	0.137	9
2021	S2	1.345	0.521	0.689	0.166	47
2021	L8	1.998	0.569	0.684	0.167	9
2021	L89 †	1.998	0.569	0.684	0.167	9
2022	S2	2.662	1.615	0.803	0.158	28
2022	L8	8.348	1.556	0.807	0.158	9
2022	L9	16.189	1.951	0.803	0.159	10
2022	L89	10.783	1.781	0.804	0.159	19
2023	S2	2.345	1.541	0.907	0.130	27
2023	L8	9.992	1.916	0.901	0.134	10
2023	L9 ‡	0.370	-0.513	—	—	10
2023	L89	259.581	3.874	0.886	0.144	20
2024	S2	2.748	1.875	0.747	0.185	55
2024	L8	34.832	2.915	0.739	0.188	10
2024	L9	8.828	1.865	0.747	0.185	8
2024	L89	21.272	2.584	0.741	0.187	18
2025	S2	1.689	1.075	0.920	0.118	28
2025	L8	3.592	1.195	0.919	0.119	7
2025	L9	3.240	1.038	0.921	0.117	8
2025	L89	3.227	1.060	0.921	0.117	15

† In 2020 and 2021, Landsat 9 (L9) was not yet operational (L9 launched 27 September 2021); the L89 configuration is therefore equivalent to L8 alone for these two seasons and parameters are identical.

‡ The 2023 L9 single-sensor calibration yielded a negative fitted exponent (b = −0.513), indicating model failure (Kc decreasing with increasing NDVI). This configuration was flagged and excluded from all aggregated analyses (see Figure S1). Validation R^2 and RMSE are not reported (—).

Abbreviations: NDVI, Normalized Difference Vegetation Index; RMSE, root mean squared error (dimensionless, Kc units); R^2, coefficient of determination; S2, Sentinel-2; L8, Landsat 8; L9, Landsat 9; L89, combined Landsat 8+9 configuration; ODP, Hungarian National Meteorological Service (OMSZ) agrometeorological data provider.

Table S2. Annual leave-one-year-out cross-validation R^2 by Kc calibration method for the Sentinel-2 (S2) configuration, corrected pipeline. The Rescale method implements a linear NDVI-to-Kc rescaling to the FAO-56 tabulated trapezoidal Kc range; the Power

method fits the nonlinear relationship Kc = a·NDVI^b (parameters in Table S1). All six growing seasons (2020–2025) are included. Values ≥ 0.80 indicate good predictive agreement with in situ ETc observations.

Year	Rescale R^2	Power R^2
2020	0.777	0.795
2021	0.436	0.689
2022	0.732	0.803
2023	0.842	0.907
2024	0.547	0.747
2025	0.852	0.920

Abbreviations: R^2, coefficient of determination; Rescale, linear NDVI-to-Kc rescaling method; Power, power-law Kc = a·NDVI^b calibration method; S2, Sentinel-2.

Table S3. Sensor configuration performance summary across six growing seasons (2020–2025), corrected pipeline. Pixel Regression R^2 is the median annual R^2 between the spatially distributed fused-NDVI values and the in situ Kc proxy (ETc/ET_0) at image-pixel resolution, reflecting the spatial coherence of the NDVI–Kc relationship. Power Kc Validation R^2 is the mean annual leave-one-year-out cross-validation R^2 (Sentinel-2 used as ETc reference baseline). Note: Landsat 9 (L9) values are averaged over 2022–2025 only; the 2023 L9 failure year is excluded from the L9 and L89 validation mean.

Sensor	Pixel Regression R^2 (median, across years)	Power Kc Validation R^2 (mean, across years)
S2	0.499	0.810
L8	0.494	0.812
L9	0.374	0.618
L89	0.453	0.810

Abbreviations: S2, Sentinel-2; L8, Landsat 8; L9, Landsat 9; L89, combined Landsat 8+9; R^2, coefficient of determination; ETc, crop evapotranspiration; ET_0, reference evapotranspiration; Kc, crop coefficient; NDVI, Normalized Difference Vegetation Index.

Table S4. Seasonal ET_0 and hydroclimatic summary for six maize growing seasons (2020–2025) at ODP agrometeorological station 73505, Nyírlugos (47.88° N, 22.32° E), Hungary. Growing seasons span approximately May–September, bounded by planting and harvest dates. ET_0 was computed following FAO-56 Penman–Monteith guidelines (Allen et al., 1998). Heat-stress days are defined as days on which daily maximum temperature ($T_{ma}{}^{x}$) ≥ 35 °C. Precipitation (P) is the cumulative in-season rainfall measured at station 73505.

Year	ET_0 (mm)	ET_0 Source	P (mm)	Days T ≥ 35°C
2020	400	ODP + Davis Vantage Pro2 (in situ)	386	0
2021	297	ODP + Davis Vantage Pro2 (in situ)	212	5
2022	618	ODP (OMSZ synoptic)	211	11
2023	546	ODP (OMSZ synoptic)	254	2
2024	588	ODP (OMSZ synoptic)	253	10
2025	566	ODP (OMSZ synoptic)	213	3

Abbreviations: ET_0, Penman–Monteith reference evapotranspiration (FAO-56); P, growing-season cumulative precipitation; $T_{ma}{}^{x}$, daily maximum air temperature; ODP, OMSZ agrometeorological data provider; Davis, Davis Vantage Pro2 automatic weather station co-located with the experimental field.

SUPPLEMENTARY FIGURES

Figure S1. *Power-law crop coefficient (Kc) calibration curves (Kc = a·NDVI^b) by sensor and growing season for the corrected pipeline (positive-b constraint enforced for Sentinel-2). Each panel corresponds to one growing season (2020–2025). Solid curves represent the fitted NDVI–Kc relationship per sensor: Sentinel-2 (S2; green), Landsat 8 (L8; orange), Landsat 9 (L9; red), and combined Landsat*

8+9 (L89; purple). In 2020 and 2021, L9 was not yet operational; L8 and L89 curves therefore overlap. The dashed red curve labelled △ in the 2023 panel denotes the rejected L9 configuration (fitted b = −0.51; $R^2 \approx 0.00$), which was excluded from all aggregated analyses. All curves are hard-capped at Kc = 1.50, consistent with the FAO-56 upper limit for maize under optimal growing conditions. Legend entries report the fitted exponent (b) and leave-one-year-out cross-validation R^2 for each sensor–year combination. Full calibration parameters are provided in Table S1.

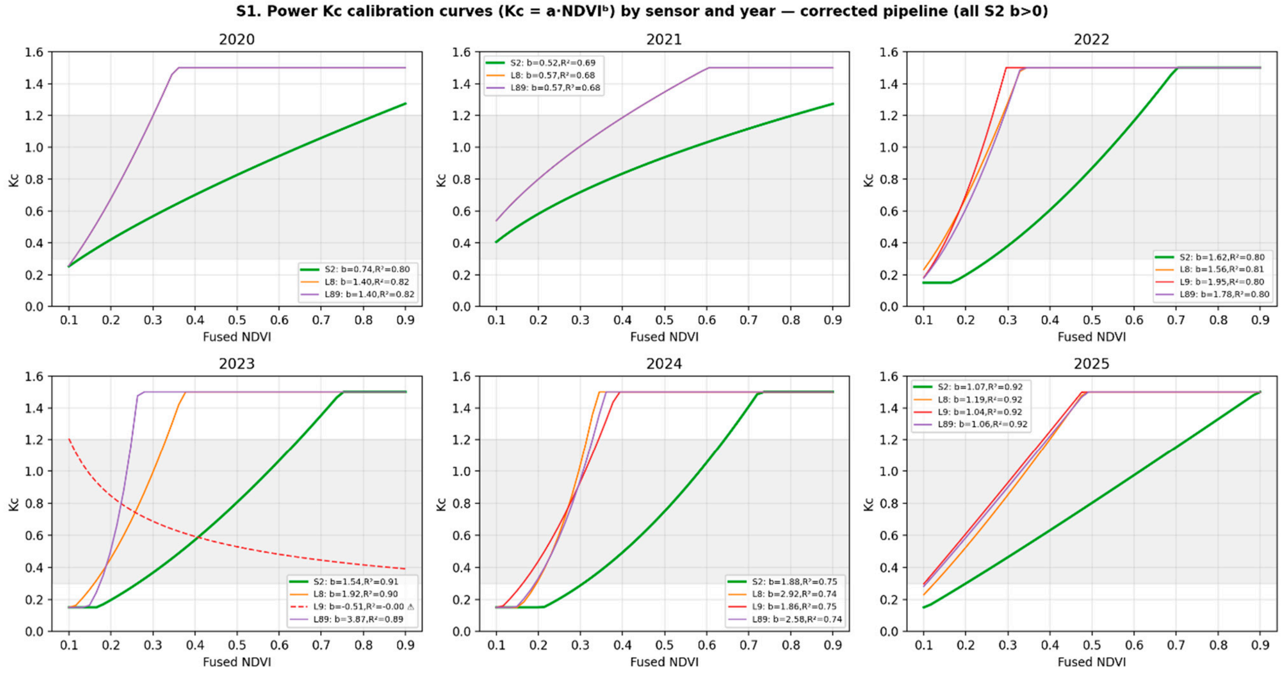

NDVI: Normalized Difference Vegetation Index; Kc: crop coefficient.

Figure S2. *Daily reference evapotranspiration (ET_0; mm d^{-1}) over the six maize growing seasons (2020–2025) at ODP agrometeorological station 73505, Nyírlugos. Panel titles report the cumulative growing-season ET_0 (mm) and the meteorological data source. For 2020 and 2021, ET_0 was computed from combined ODP synoptic records and measurements from an on-site Davis Vantage Pro2 automatic weather station (ODP + Davis); for 2022–2025, ODP records alone were used following hardware upgrades to the primary station. All ET_0 values were calculated by the FAO-56 Penman–Monteith equation (Allen et al., 1998). Season-level ET_0 totals and data source metadata are tabulated in Table S4. The pronounced inter-annual variability (range: 297–618 mm) reflects contrasting hydroclimatic regimes across the study period, with 2022 being an exceptional drought year (cumulative ET_0 = 618 mm) and 2021 a mild, frequently overcast season (297 mm).*

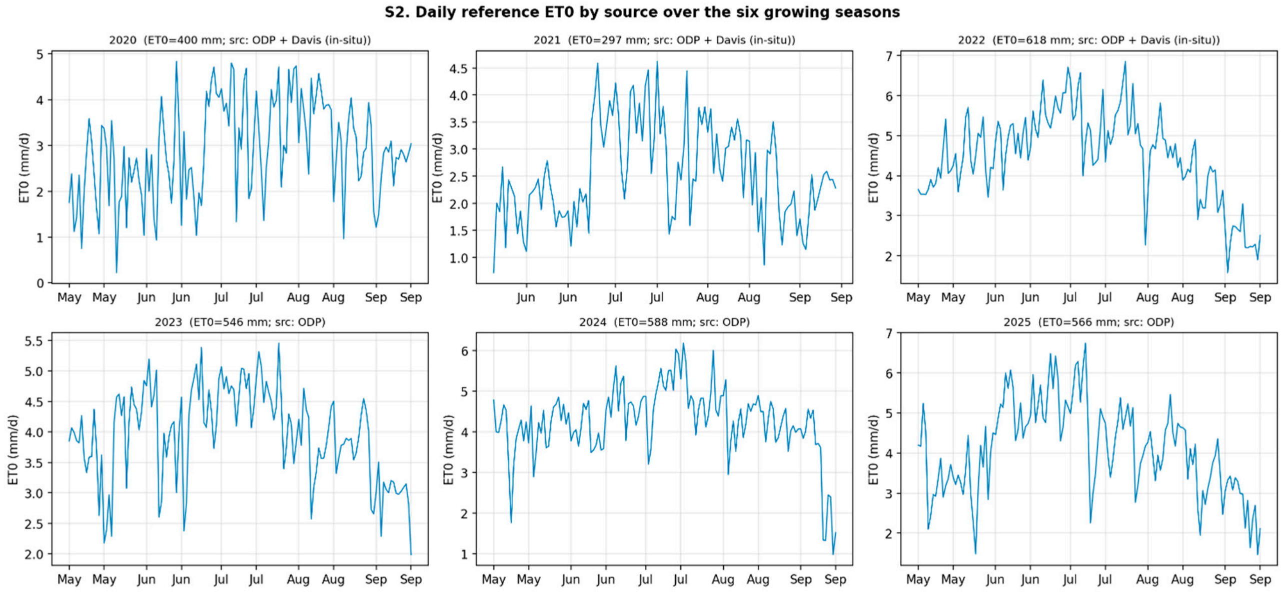

ODP: OMSZ agrometeorological data provider; ET_0: reference evapotranspiration.

Figure S3. *Leave-one-year-out cross-validation R^2 distributions by Kc calibration method across six growing seasons (2020–2025) for Sentinel-2 (S2), corrected pipeline. Box boundaries represent the interquartile range (IQR; 25th–75th percentile); the orange horizontal line indicates the median; whiskers extend to 1.5 × IQR. Results are shown for four method–filter combinations: Rescale (all)—linear NDVI-to-Kc rescaling applied regardless of the sign of the calibration slope; Power (all)—power-law fit applied to all years; Rescale (positive)—restricted to years with a positive rescaling slope; Power (positive)—restricted to years in which the fitted exponent $b > 0$. Green boxes indicate Power-law configurations; red boxes indicate Rescale configurations. The Power-law method (both filter variants) consistently yields higher median R^2 and a narrower interquartile range than the corresponding Rescale variant, supporting the adoption of the Power-law approach as the primary Kc estimator. The dotted horizontal line at $R^2 = 0$ is shown for reference. Annual R^2 values underlying the distributions are tabulated in Table S2.*

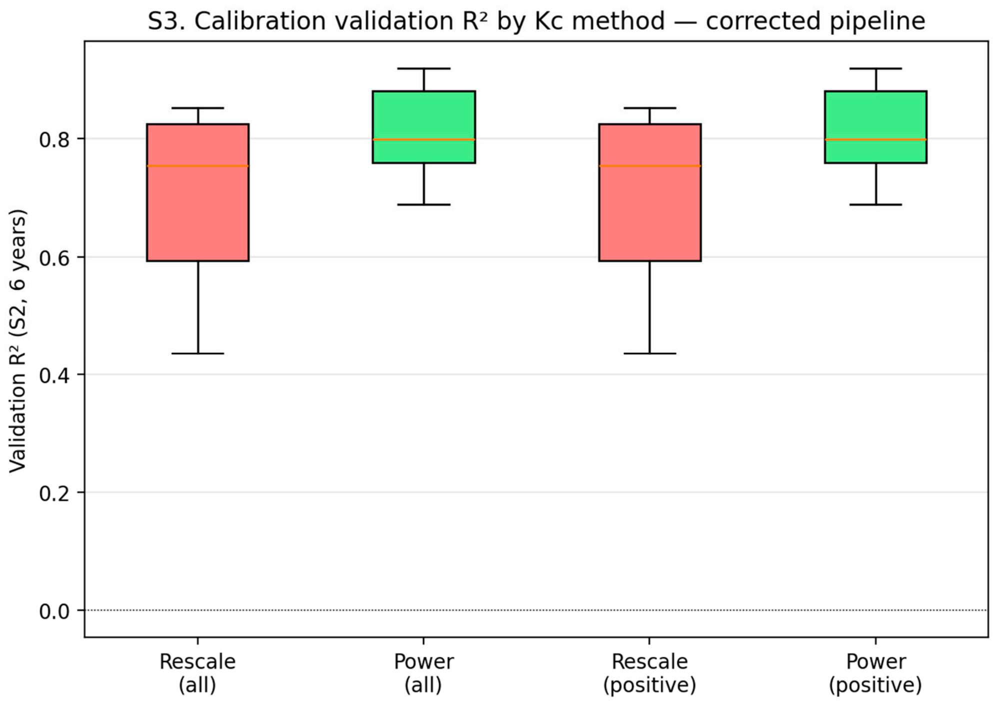

[

R^2: coefficient of determination; IQR: interquartile range; Kc: crop coefficient; NDVI: Normalized Difference Vegetation Index.

Figure S4. *Sensor configuration performance summary across six growing seasons (2020–2025), corrected pipeline. Blue bars represent the median annual pixel-level regression R^2 between spatially distributed fused-NDVI values and the in situ Kc proxy (ET_c/ET_0), measuring the spatial coherence of the satellite-derived NDVI–Kc relationship within each growing season. Green bars represent the mean annual Power-law Kc validation R^2 from leave-one-year-out cross-validation, using Sentinel-2 (S2) ETc as the reference. Sensor configurations: Sentinel-2 (S2), Landsat 8 (L8), Landsat 9 (L9), and combined Landsat 8+9 (L89). S2 and L8 achieve comparable validation R^2 (≈ 0.81), whereas L9 underperforms (mean validation $R^2 = 0.62$), consistent with the higher proportion of anomalous calibrations in that sensor's record (2023 failure; see Table S1). The divergence between pixel regression R^2 (spatial) and validation R^2 (temporal) highlights that spatial NDVI heterogeneity does not necessarily translate directly into temporal Kc predictive performance. Numerical values are tabulated in Table S3.*

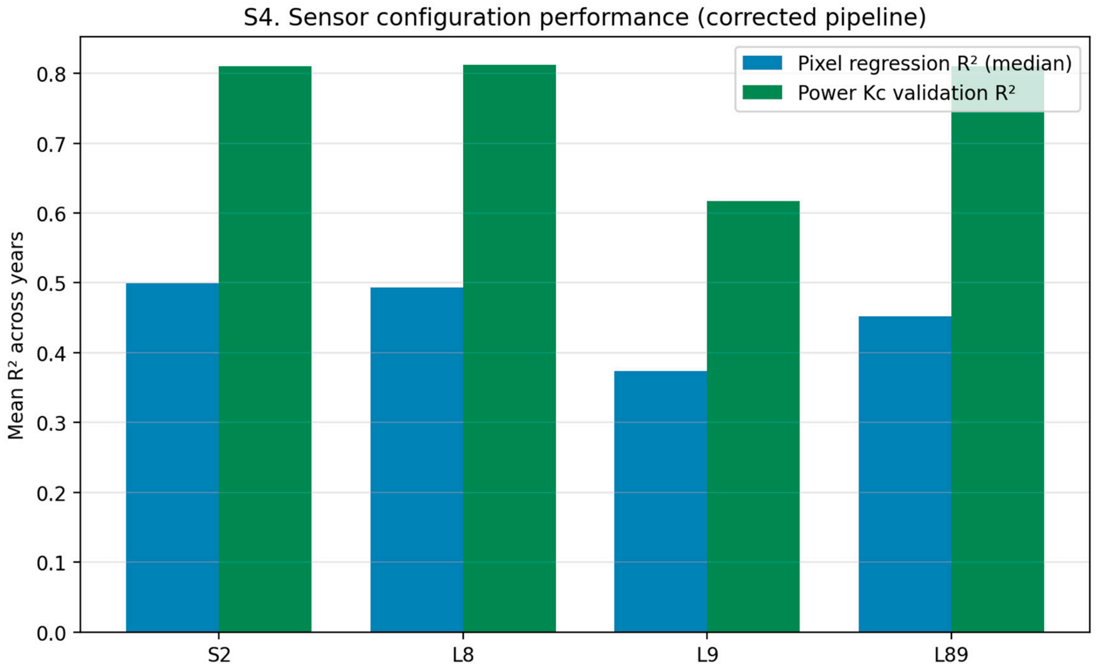

R^2: coefficient of determination; Kc: crop coefficient; ETc: crop evapotranspiration; ET_0: reference evapotranspiration; NDVI: Normalized Difference Vegetation Index.

Figure S5. *Hydroclimatic conditions over six maize growing seasons (2020–2025) at ODP agrometeorological station 73505, Nyírlugos (47.88° N, 22.32° E). Red line (left axis): daily maximum air temperature (T_{ma}^{x}, °C). Blue stepped line (right axis): cumulative growing-season precipitation (P, mm). The horizontal dashed grey line marks the 35 °C heat-stress threshold for maize; an additional dashed red line at 35 °C is shown in the 2024 panel for visual reference given the sustained near-threshold temperatures recorded that season. Panel titles report total growing-season precipitation (mm) and the number of heat-stress days ($T_{ma}^{x} \geq 35$ °C) per year. The six seasons encompass a broad hydroclimatic gradient: from the well-watered 2020 season (P = 386 mm; zero heat-stress days) to the severe drought of 2022 (P = 211 mm; 11 heat-stress days). Seasons 2021–2025 all experienced measurable precipitation deficits (minimum cumulative P–ETc ranging from −85 to −343 mm; see Figure S6). Aggregated hydroclimatic statistics are tabulated in Table S4.*

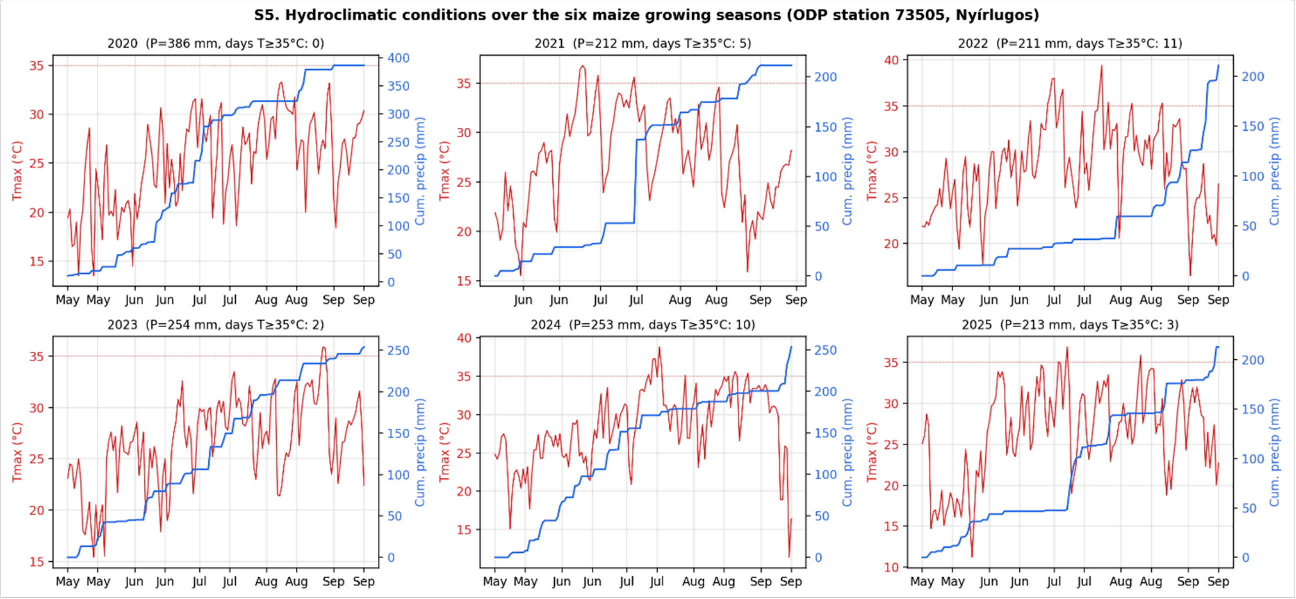

ODP: OMSZ agrometeorological data provider; T_{ma}^{x}: daily maximum temperature; P: cumulative precipitation.

Figure S6. *Cumulative daily meteorological water balance (P – ETc, mm) over the six maize growing seasons (DOY 120–260), computed using the Power-law Kc method with Sentinel-2 as the primary data source. The x-axis represents day of year (DOY); the y-axis is the cumulative P – ETc balance. Positive values indicate a meteorological surplus (in-season precipitation exceeds remotely sensed crop water demand); negative values indicate a deficit requiring supplemental irrigation to avoid crop water stress. The dashed horizontal line at 0 mm delineates surplus from deficit conditions. Legend entries report the annual minimum cumulative P – ETc (mm), representing the maximum observed seasonal water deficit. Only 2020 avoided a sustained negative balance (minimum = +7 mm), while 2022 reached a peak deficit of −343 mm. The trajectory differences reflect both the precipitation variability documented*

in Figure S5 and the elevated ET_0 in high-radiation years (Table S4). Underlying daily P – ETc series are provided in the companion data file (S6_cumulative_deficit.csv).

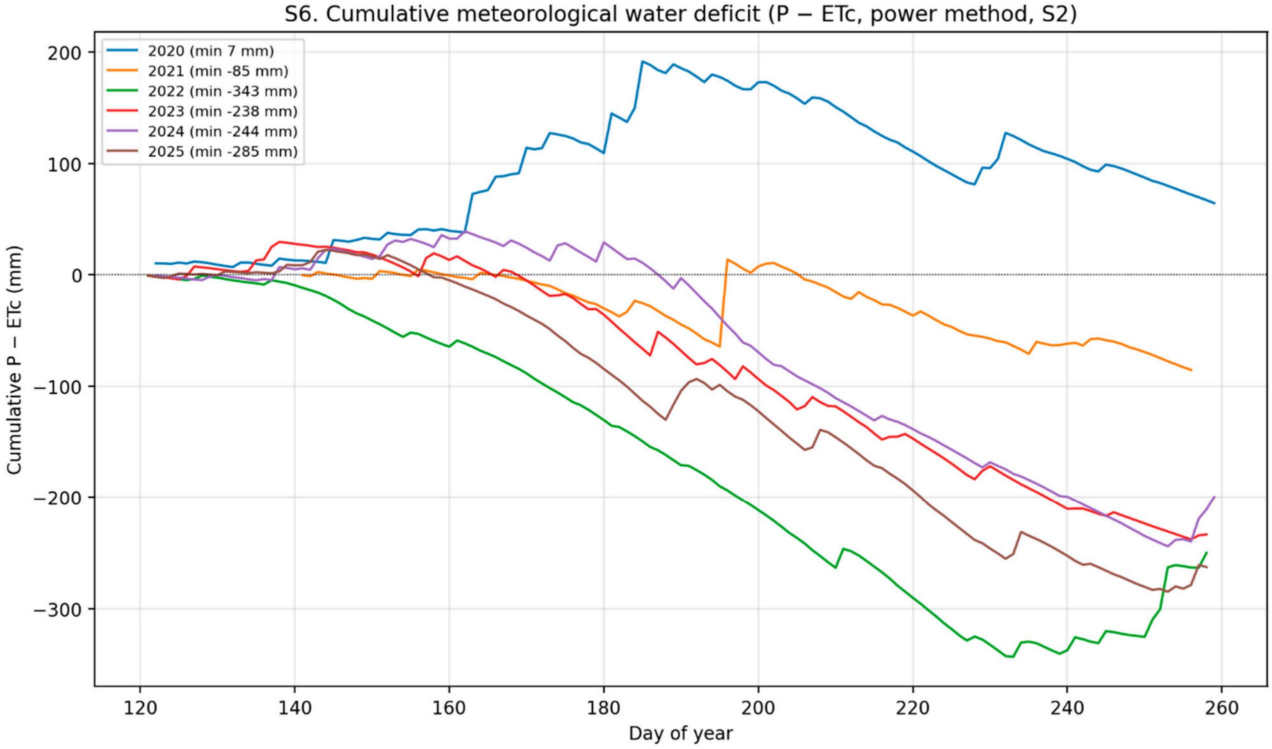

ETc: crop evapotranspiration (Power-law Kc method, Sentinel-2); P: daily precipitation; DOY: day of year; Kc: crop coefficient.

SUPPLEMENTARY DATA FILES

The following electronic supplementary data files are available from the authors upon reasonable request.

1. **CURRENT_calibration_params_all_configs.csv** — Full Kc calibration parameter table for all sensor–year combinations (power-law and rescale methods), including pixel regression R^2, mean slope, and the number of Landsat and Sentinel-2 dates per season. Underlies Table S1.
2. **CURRENT_et0_crossval_daily_2020_2021.csv** — Daily ET_0 values (mm d^{-1}) from both the MATLAB reference implementation and the Python processing pipeline for growing seasons 2020 and 2021.
3. **daily_series_S2_2020-2025.csv** — Full daily time series for growing seasons 2020–2025 (Sentinel-2 configuration), including MODIS reference NDVI, fused-NDVI median, Kc (Rescale and Power methods), ETc (both methods), ET_0, and daily precipitation.
4. **S3_validationR2_by_method.csv** — Annual leave-one-year-out cross-validation R^2 by Kc method (Rescale and Power) for Sentinel-2. Underlies Table S2 and Figure S3.
5. **S4_config_performance.csv** — Sensor configuration performance metrics (pixel regression R^2 and Power Kc validation R^2). Underlies Table S3 and Figure S4.
6. **S6_cumulative_deficit.csv** — Daily cumulative meteorological water balance (P – ETc, mm) by DOY for all six growing seasons. Underlies Figure S6.
7. **GEE reprocubility scripts**

Document generated from original supplementary materials for the Sensors (MDPI) publication: "Multi-Sensor NDVI-Based Crop Coefficient Estimation for Maize Evapotranspiration via MODIS–Sentinel-2 Fusion: A Six-Year Field Evaluation in North-East Hungary" | sensors-4362312
